# Kähler-Einstein Metrics

**DOI:** 10.1007/s12220-026-02401-4

**Published:** 2026-03-19

**Authors:** Friedrich Haslinger

**Affiliations:** Fakultät für Mathematik, Universität Wien, Oskar-Morgenstern-Platz 1, A-1090 Wien, Austria

**Keywords:** Kähler-Einstein metrics, Real holomorphic vector fields, Primary 32Q15, 32Q20, Secondary 53C50

## Abstract

We recall a simple formula for a Kähler-Einstein metric on the unit ball and on the Siegel upper half space, both together with real holomorphic vector fields and consider generalized complex ellipsoids in $\mathbb {C}^n$ and show that the logarithm of the defining function, as a potential function, provides a pseudometric, which is Kähler-Einstein. In addition we prove that the complex ellipsoids, endowed with this pseudometric have a real holomorphic vector field, which has several far-reaching differential geometric and functional analytic consequences. Finally we give an example of a real holomorphic vector field of higher order.

## Introduction

S. Y. Cheng and S.T. Yau [3] showed that any $\mathcal {C}^2$ bounded pseudoconvex domain in $\mathbb {C}^n$ has a complete Kähler-Einstein metric with negative Ricci curvature. Their metric is given by $(h_{j \bar{k}})= (\frac{\partial ^2 h}{\partial z_j \partial \bar{z}_k}),$ where *h* satisfies the Monge-Ampere equation $\det [\frac{\partial ^2 h}{\partial z_j \partial \bar{z}_k}]= e^{(n+1)h},$ with $h =\infty$ at the boundary. N. Mok and S.T. Yau [15] have extended this result to arbitrary bounded pseudoconvex domains in $\mathbb {C}^n.$ An equivalent description is to find a solution of the equation $$(-1)^n \, {\text {det}}\begin{pmatrix} u &  u_{\bar{k}} \\ u_j &  u_{j\bar{k}} \end{pmatrix} =1$$ in $\Omega ,$ and $u=0$ at $b\Omega .$ If one takes for $h=-\log u,$ we get the solution from before. Explicit solutions are only known in the simplest cases. If $\Omega$ is the unit ball, then the hyperbolic metric is the Bergman metric, which is also Kähler-Einstein. There are also formulas for more general domains, see [4] and [5], so one can compute the Bergman metric. The resolution of Cheng’s Conjecture [7, 11, 14] characterizes the unit ball in $\mathbb {C}^n$, up to biholomorphisms, as the only smoothly bounded strictly pseudoconvex domain in $\mathbb {C}^n$ with a Bergman metric that is also Kähler-Einstein, which is also true for smoothly bounded pseudoconvex domains in $\mathbb {C}^2$ of finite type ([17]). The study of singular Kähler-Einstein metrics has attracted significant interest in complex geometry and geometric analysis.

Here, we will concentrate on the question, whether there is a way of constructing a Kähler-Einstein metric at least on a large subset of a Kähler manifold, see also [16]. The purpose of this paper is to give a simple explicit pseudometric for $\Omega$ both being Kähler-Einstein and having a real holomorphic vector field, i.e. a smooth real valued function $\psi :\Omega \longrightarrow \mathbb {R}$ such that $h^{j \bar{k}} \psi _{\bar{k}}$is holomorphic for each $j=1,\dots ,n,$ where $(h^{j \bar{k}})$ is the transpose of the inverse matrix of $(h_{j \bar{k}}),$ this means there exists a function with real holomorphic vector field, which is related to Calabi’s extremal Kähler metric [2]. This property also holds for the unit ball endowed with the hyperbolic metric, see [9, 10, 13]. The real holomorphicity of the gradient vector field of a weight function implies Liouville theorems for weighted holomorphic functions, see [13].

Let $A^2(\mathbb {C}^n, e^{-|z|^2})$ denote the Segal Bargmann space of entire functions *f*,  where $\mathbb {C}^n$ carries the Euclidean metric and $\int _{\mathbb {C}^n} |f(z)|^2 e^{-|z|^2}\, d\lambda (z)< \infty .$ The function $\psi (z)=|z|^2$ yields a real holomorphic vector field, we have in this case $h^{j \bar{k}} \psi _{\bar{k}}=z_j;$ the adjoint of the differentiation operator $\frac{\partial f}{\partial z_j}$ on $A^2(\mathbb {C}^n, e^{-|z|^2})$ is the multiplication operator $z_j f,$ both being unbounded densely defined operators on $A^2(\mathbb {C}^n, e^{-|z|^2}),$ in quantum mechanics they are the annihilation and creation operators, see [8].

For the main result of this paper we consider consider generalized complex ellipsoids and show that there exists a smooth Kähler-Einstein metric away from an analytic subvariety, the metric is simply given by $-\partial \bar{\partial } \log (-\rho ),$ where $\rho$ is the defining function of the domain. In addition we point out how to find examples with real holomorphic vector fields where $h^{j \bar{k}} \psi _{\bar{k}}$ is holomorphic of higher order.

## Kähler-Einstein Metric for the Unit Ball and the Siegel-Upper Halfspace

The hyperbolic metric on the unit ball $\mathbb {B} \subset \mathbb {C}^n$ is given by the potential function

$$\begin{aligned} h = -\log (1-|z|^2), \end{aligned}$$

and we get

$$\begin{aligned} h_{j \bar{k}}=(1-|z|^2)^{-1} \delta _{jk} + (1-|z|^2)^{-2} \bar{z}_j z_k, \end{aligned}$$

where $|z|^2= \sum _{j=1} |z_j|^2;$ for the transpose of the inverse we obtain

$$\begin{aligned} h^{j \bar{k}}=(1-|z|^2) (\delta _{jk} - z_j \bar{z}_k ), \end{aligned}$$

and the determinant is

$$\begin{aligned} {\text {det}}(h_{j \bar{k}}) = (1-|z|^2)^{-n-1}. \end{aligned}$$

The Ricci curvature of $(h_{j \bar{k}})$ is given by

$$\begin{aligned} {\text {Ric}}_{j \bar{k}} = - \frac{\partial ^2 }{\partial z_j \partial \bar{z}_k} \, \log \,{\text {det}}(h_{j \bar{k}}). \end{aligned}$$

Hence

$$\begin{aligned} {\text {Ric}}_{j \bar{k}} =-(n+1) h_{j \bar{k}}, \end{aligned}$$

and hence $(h_{j \bar{k}})$ is Kähler -Einstein on $\mathbb {B},$ the scalar curvature $\lambda$ equals $-(n+1).$ It is also the Bergman-Kähler metric, as the Bergman kernel of $\mathbb {B}$ is

$$\begin{aligned} B(z,z)= \frac{n!}{\pi ^n}(1-|z|^2)^{-n-1}. \end{aligned}$$

This is one of the few examples where the Kähler-Einstein metric can be computed explicitly.

If we choose $\psi (z) = (1-|z|^2)^{-1},$ we get the real holomorphic vector field

$$\begin{aligned} h^{j\bar{k}} \psi _{\bar{k}} =z_j; \end{aligned}$$

and the adjoint operator of $\partial$ on the weighted Bergman space of holomorphic functions

$$A^2 (\mathbb {B}, h, d\mu ) = \{ f: \mathbb {B} \longrightarrow \mathbb {C} : \int _{\mathbb {B}} |f(z)|^2 \, (1-|z|^2)^{-n-1} \, \exp {(-(1-|z|^2)^{-1})} \, d\lambda (z) < \infty \}$$

is the multiplication operator

$$\begin{aligned} \partial ^* (u^j \,dz_j) = z_j u^j. \end{aligned}$$

For further details see [8].

J.S. Bland [1] described the Kähler-Einstein metric for the domain $\Omega$, where $p_1= \dots = p_{n-1}=1, p_n =p,$ for $p>1,$ exhibiting a wide range of boundary behavior. Isaev [12] generalized Bland’s description. More recently, P. Ebenfelt, Ming Xiao and Hang Xu extended Bland’s construction to a broader setting, see Theorem 1.4 in [6].

Next we show that the Siegel-upper halfspace

$$\mathcal {U} := \{ (z_1, z_2, \dots z_n, z_{n+1})\in \mathbb {C}^{n+1} : \Im z_{n+1} > \sum _{j=1}^n |z_j|^2 \} $$

also has Kähler-Einstein metric with a real holomorphic vector field. For this purpose we set

$$\begin{aligned} h = - \log ( \Im z_{n+1} - \sum _{j=1}^n |z_j|^2 ). \end{aligned}$$

Let $$N= \Im z_{n+1} - \sum _{j=1}^n |z_j|^2.$$ Then

$$\begin{aligned} h_{j\bar{j}} = \frac{N+ |z_j|^2}{N^2}, \, j=1, \dots , n \end{aligned}$$

and

$$\begin{aligned} h_{j\bar{k}}= \frac{\bar{z}_j z_k}{N^2}, \, j\ne k, \, j,k=1, \dots ,n; \end{aligned}$$

and we get

$$\begin{aligned} h_{j \overline{n+1}} = \frac{\bar{z}_j}{2i N^2}, \, j=1, \dots , n \end{aligned}$$

finally

$$\begin{aligned} h_{n+1 \overline{n+1}} = \frac{1}{4N^2}, \end{aligned}$$

in addition we have

$$\begin{aligned} h_{\bar{j} k}= \overline{ h_{j \bar{k}}}. \end{aligned}$$

First we compute the determinant and inverse of $A:=(h_{j\bar{k}})^n_{j,k=1}:$ observe that, by Sherman-Morrison, we have

$$\begin{aligned} {\text {det}}( A) = (1+V^tE^{-1}U)\, {\text {det}}(E), \end{aligned}$$

where *E* is the diagonal matrix with entries $\frac{1}{N}$ and $V^t=(\frac{z_1}{N}, \dots , \frac{z_n}{N})$ and $U^t=(\frac{\bar{z}_1}{N}, \dots , \frac{\bar{z}_n}{N}).$ Hence we get

$$\begin{aligned} {\text {det}}( A) = \frac{1}{N^n}(1+\frac{1}{N} \sum _{j=1}^n |z_j|^2). \end{aligned}$$

By Sherman-Morrison we have

$$\begin{aligned} A^{-1} = E^{-1} - \frac{1}{1+ V^t E^{-1} U}\, E^{-1}U V^t E^{-1}. \end{aligned}$$

Hence we obtain

$$A^{-1} = \frac{N}{y_{n+1}} \begin{pmatrix} y_{n+1}-|z_1|^2 &  -\bar{z}_1 z_2 &  \dots &  -\bar{z}_1 z_n \\ -\bar{z}_2 z_1 &  y_{n+1}-|z_2|^2 &  \dots &  -\bar{z}_2 z_n \\ \dots &  \dots & \dots &  \dots \\ -\bar{z}_n z_1 &  -\bar{z}_n z_2 &  \dots &  y_{n+1}-|z_n|^2 \end{pmatrix} $$

Next we use a formula for the determinant of block matrices:

$${\text {det}}((h_{j\bar{k}})^{n+1}_{j,k=1}) = {\text {det}} \begin{pmatrix} A &  B \\ C &  D \end{pmatrix} = {\text {det}}(A) \, {\text {det}}(D-CA^{-1}B),$$

where $D=\frac{1}{4N^2},$ and $B^t = \frac{1}{2i}(\frac{\bar{z}_1}{N^2}, \dots , \frac{\bar{z}_n}{N^2} )$ and $C= -\frac{1}{2i}(\frac{ z_1}{N^2}, \dots , \frac{\ z_n}{N^2} ).$ We get

$$\begin{aligned} D-CA^{-1}B = \frac{1}{4N y_{n+1}}, \end{aligned}$$

hence

1 $$\begin{aligned} {\text {det}}((h_{j\bar{k}})^{n+1}_{j,k=1})= \frac{1}{N^n}(1+\frac{1}{N} \sum _{j=1}^n |z_j|^2) \frac{1}{4N y_{n+1}}= \frac{1}{4 N^{n+2}}. \end{aligned}$$

Finally we use a formula for the inverse of a block matrix, setting $(D-CA^{-1}B)^{-1} =a$ we have

$$((h_{j\bar{k}})^{n+1}_{j,k=1})^{-1} = \begin{pmatrix} A^{-1}+ a A^{-1}BC A^{-1} &  -a A^{-1}B \\ -a CA^{-1} &  a \end{pmatrix},$$

and get

2 $$\begin{aligned} ((h_{j\bar{k}})^{n+1}_{j,k=1})^{-1} = \begin{pmatrix} N &  0 &  0 &  \dots &  0 &  2iN \bar{z}_1\\ 0 &  N &  0 &  \dots &  0 &  2iN \bar{z}_2\\ 0 &  0 &  N &  \dots &  0 &  2iN \bar{z}_3\\ \dots &  \dots &  \dots &  \dots &  \dots &  \dots \\ 0 &  0 &  0 &  0 &  N &  2iN \bar{z}_n\\ -2iN z_1 &  -2iN z_2 &  -2iN z_3 &  \dots & -2iN z_n &  4Ny_{n+1} \end{pmatrix}. \end{aligned}$$

The real valued function $\psi (z) = (y_{n+1} - \sum _{j=1}^n |z_j|^2)^{-1}$ provides a real holomorphic vector field: we denote the transpose of the inverse matrix of $(h_{j\bar{k}})^{n+1}_{j,k=1})$ by $(h^{j \bar{k}})$ and get by (2)

$$\begin{aligned} h^{j \bar{k}} \psi _{\bar{k}} = 0, \, j=1,\dots n \ \ {\text {and}} \ \ h^{n+1 \bar{k} } \psi _{\bar{k}} = 2i. \end{aligned}$$

The Ricci curvature of $(h_{j\bar{k}})^{n+1}_{j,k=1})$ is

$$\begin{aligned} {\text {Ric}}_{j \bar{k}} = - \frac{\partial ^2 }{\partial z_j \partial \bar{z}_k} \, \log \,{\text {det}}(h_{j \bar{k}}) = -(n+2) h_{j \bar{k}}, \end{aligned}$$

where we used (1), hence $(h_{j \bar{k}})$ is Kähler-Einstein and the scalar curvature equals $-(n+2).$

## Pseudometrics for General Ellipsoids

Here we replace the monomials $z_j$ by holomorphic functions $f^j (z_j).$

### Theorem 3.1

Let

$$\begin{aligned} \Omega =\{ z=(z_1,z_2, \dots , z_n)\in \mathbb {C}^n : \sum _{j=1}^n |f^j (z_j)|^2 <1 \}, \end{aligned}$$

where $f^j$ are entire holomorphic functions. Let $f^j_j$ denote the derivative of $f^j$ with respect to $z_j$ and let $\bar{f}^j_{\bar{j}}$ denote the derivative of $\bar{f}^j$ with respect to $\bar{z}_j.$ Set $N = 1-\sum _{j=1}^n |f^j (z_j)|^2$ and $h=-\log N.$ Then

3 $$\begin{aligned} {\text {det}}(h_{j \bar{k}}) = 1/N^{n+1} \, \prod _{j=1}^n |f^j_j|^2. \end{aligned}$$

and for the real valued function $\psi = N^{-1}$ we get the real holomorphic vector field

$$\begin{aligned} h^{j, \bar{k}} \psi _{\bar{k}} = \frac{f^j}{f^j_j}, \end{aligned}$$

$$j=1, \dots ,n.$$

### Proof

Note that

$$\begin{aligned} |f^j _j|^2 = f^j_j \bar{f}^j_{\bar{j}}. \end{aligned}$$

Then we have

$$h_{j \bar{j}}= \frac{1}{N^2} \, (|f^j_j|^2(N+ |f^j|^2 )) , \ \ h_{j \bar{k}}= \frac{1}{N^2} \, f^j_j \bar{f}^j f^k \bar{f}^k_{\bar{k}},$$

for $$j\ne k.$$

In order to compute the transpose of the inverse of $[h_{j \bar{k}}]$ we use the Sherman-Morrison formula

$$\begin{aligned} (A+UV^t)^{-1} = A^{-1} - \frac{1}{1+V^t A^{-1} U} \,\, A^{-1} U V^t A^{-1}, \end{aligned}$$

where *A* is an invertible $n\times n$-matrix and *U* and *V* are vectors in $\mathbb {C}^n.$

In our case we take *A* as the diagonal matrix with entries $|f^j_j|^2 /N$ for $j=1, \dots , n.$

We define

$$U^t= \big ( \frac{1}{N} f^1_1 \bar{f}^1, \frac{1}{N} f^2_2 \bar{f}^2, \dots , \frac{1}{N} f^n_n \bar{f}^n \big ), \ \ V^t = \big ( \frac{1}{N} f^1 \bar{f}^1_{\bar{1}}, \frac{1}{N} f^2 \bar{f}^2_{\bar{2}}, \dots , \frac{1}{N} f^n \bar{f}^n_{\bar{n}} \big ).$$

A straightforward computation shows

$$\begin{aligned} 1+V^t A^{-1} U = \frac{1}{N} \end{aligned}$$

and, since

$$\begin{aligned} {[}h^{j \bar{k}}] = ((A+ UV^t)^{-1})^t \end{aligned}$$

we derive from the Sherman-Morrison formula that

$$h^{j,\bar{j}} = \frac{N -N |f^j|^2}{|f^j_j|^2} \, \ , \ h^{j, \bar{k}} = - \frac{N f^j \bar{f}^k}{\bar{f}^k_{\bar{k}} f^j_j}.$$

The real valued function $\psi = N^{-1}$ provides a real holomorphic vector field: we have

$$\begin{aligned} \psi _{\bar{k}} = \frac{f^k \bar{f}^k _{\bar{k}}}{N^2} \end{aligned}$$

and

4 $$\begin{aligned} h^{1, \bar{k}} \psi _{\bar{k}}= &   \frac{1}{N |f^1_1|^2} [ (1-|f^1|^2) f^1 \bar{f}^1_{\bar{1}} - f^2 \bar{f}^2 f^1 \bar{f}^1_{\bar{1}}- \dots - f^n \bar{f}^n f^1 \bar{f}^1_{\bar{1}}]\nonumber \\= &   \frac{1}{N f^1_1}[ f^1(1-|f^1|^2- \dots - |f^n|^2)] \nonumber \\= &   \frac{f^1}{f^1_1} \end{aligned}$$

similarly

$$\begin{aligned} h^{j, \bar{k}} \psi _{\bar{k}} = \frac{f^j}{f^j_j}, \end{aligned}$$

for $$j=2, \dots ,n.$$

For the determinant of $(h_{j \bar{k}})$ we get from Sherman-Morrison that

$$\begin{aligned} {\text {det}}(h_{j \bar{k}}) = {\text {det}}(A+UV^t)= (1+V^tA^{-1}U)\, {\text {det}}(A), \end{aligned}$$

and obtain

$$\begin{aligned} {\text {det}}(h_{j \bar{k}}) = 1/N^{n+1} \, \prod _{j=1}^n |f^j_j|^2. \end{aligned}$$

$$\square $$

### Remark 3.2

Since the determinant can become zero, we only have a pseudometric on $\Omega .$ The Ricci curvature of $h=(h_{j \bar{k}})$ is given by

$$\begin{aligned} {\text {Ric}}_{j \bar{k}} = - \frac{\partial ^2 }{\partial z_j \partial \bar{z}_k} \, \log \,{\text {det}}(h_{j \bar{k}}). \end{aligned}$$

Since the function $z_j \mapsto \log |f^j_j(z_j)|^2$ is harmonic for $f^j_j(z_j)\ne 0,$ we get

$$\begin{aligned} {\text {Ric}}_{j \bar{k}} =-(n+1) h_{j \bar{k}} \end{aligned}$$

hence $(h_{j \bar{k}})$ is Kähler -Einstein on $\Omega \setminus D$ and the scalar curvature $\lambda$ equals $-(n+1),$ where *D* is the divisor defined by $f^j_j (z_j)=0, j=1, \dots , n.$

**Examples**

**(a)**: If $f^j(z_j)=z_j^{p_j},$ for $p_j \in \mathbb {N}, j=1,\dots ,n,$ we get the complex ellipsoid $\Omega$ in $\mathbb {C}^n$ with defining function

$$\begin{aligned} \rho (z)= -1+ \sum _{j=1}^n |z_j|^{2p_j}, \end{aligned}$$

where $$p_j \in \mathbb {N}, j=1,\dots ,n.$$

Let $$h= -\log (-\rho )$$ and $$N=1- \sum _{j=1}^n |z_j|^{2p_j}.$$

From (3) we get for the determinant

$$\begin{aligned} {\text {det}}(h_{j \bar{k}}) = 1/N^{n+1} \, \prod _{j=1}^n p_j^2 \,|z_j|^{2p_j -2}, \end{aligned}$$

and notice that the determinant equals zero for $z\in D,$ where

$$\begin{aligned} D= \{ z=(z_1, \dots ,z_n) \in \Omega : z_j = 0 \, \, {\text {if}}\, \, p_j>1\}, \end{aligned}$$

which is a divisor on $\Omega ,$ see [16] for a similar situation.

The Ricci curvature of $(h_{j \bar{k}}),$ on $\Omega \setminus D,$ is

$$\begin{aligned} {\text {Ric}}_{j \bar{k}} =-(n+1) h_{j \bar{k}}. \end{aligned}$$

The real-valued function $\psi (z) =1/(1-\sum _{j=1}^n |z_j|^{2p_j})$ provides a real holomorphic vector field: we have

$$\begin{aligned} h^{j \bar{k}} \psi _{\bar{k}} = \frac{z_j}{p_j}, \end{aligned}$$

for $$j=1, \dots , n.$$

In the following examples we obtain true Kähler-Einstein metrics.

**(b)**: Let

$$\Omega =\{ (z_1,z_2,\dots , z_n)\in \mathbb {C}^n : |e^{z_1}|^2 +|e^{z_2}|^2 + \dots + |e^{z_n}|^2 <1 \}.$$

Set $\rho =-1+ \sum _{j=1}^n |e^{z_j}|^2.$ For $N=-\rho$ and $h= - \log (-\rho )$ we get from(3) that

$$\begin{aligned} {\text {det}}(h_{j \bar{k}}) = 1/N^{n+1} \, |\exp (\sum _{j=1}^n z_j )|^2, \end{aligned}$$

we have a Kähler-Einstein metric and the real valued function $\psi (z) =1/(1-\sum _{j=1}^n |e^{z_j}|^2)$ yields the real holomorphic vector field

$$\begin{aligned} h^{j \bar{k}} \psi _{\bar{k}} = 1, \end{aligned}$$

for $$j=1, \dots , n,$$ see (4).

**(c)**: Suppose that $f^1 (z_1) = \exp (-\frac{1}{z_1}),$ for $z_1 \in \mathbb {C}^*= \mathbb {C} \setminus \{0 \}.$ Let

$$\begin{aligned} \Omega = \{ (z_1, z_2, \dots , z_n) \in \mathbb {C}^* \times \mathbb {C} \dots \times \mathbb {C} : |f^1(z_1)|^2 + \sum _{j=2}^n |z_j|^2 <1 \}. \end{aligned}$$

Now we use (4) and compute

$$\begin{aligned} h^{1 \bar{k}} \psi _{\bar{k}}= \frac{f^1(z_1)}{f^1_1(z_1)}= \frac{\exp (-\frac{1}{z_1})}{\exp (-\frac{1}{z_1})\frac{1}{z_1^2}} = z_1^2, \, h^{j \bar{k}} \psi _{\bar{k}} =z_j, \, j=2, \dots , n \end{aligned}$$

getting a real holomorphic vector field of order 2 and we have a Kähler-Einstein metric on $\Omega .$

## Data Availability

Not applicable.
